# Epidermal growth factor receptor inhibitor ameliorates excessive astrogliosis and improves the regeneration microenvironment and functional recovery in adult rats following spinal cord injury

**DOI:** 10.1186/1742-2094-11-71

**Published:** 2014-04-05

**Authors:** Zai-Wang Li, Ji-Jun Li, Lan Wang, Jian-Ping Zhang, Jing-Jing Wu, Xu-Qiang Mao, Guo-Feng Shi, Qian Wang, Feng Wang, Jian Zou

**Affiliations:** 1 Department of Neurology, Wuxi People’s Hospital of Nanjing Medical University, Wuxi, PR China; 2 Department of Clinical Laboratory Science, Wuxi People’s Hospital of Nanjing Medical University, Wuxi, PR China; 3 Department of Integrative Medicine, Shanghai Children’s Medical Center, Shanghai Jiaotong University School of Medicine, Shanghai 200127, PR China; 4 Department of Neurology, The 9th People’s Hospital of Wuxi, Hospital of Hand Surgery, Jiangsu, PR China; 5 Wuxi Clinical Science Research Institute, Wuxi, PR China

**Keywords:** Epidermal growth factor receptor, Astrogliosis, Spinal cord injury, Regeneration microenvironment

## Abstract

**Background:**

Astrogliosis is a common phenomenon after spinal cord injury (SCI). Although this process exerts positive effects on axonal regeneration, excessive astrogliosis imparts negative effects on neuronal repair and recovery. Epidermal growth factor receptor (EGFR) pathway is critical to the regulation of reactive astrogliosis, and therefore is a potential target of therapeutics to better control the response. In this report, we aim to investigate whether blocking EGFR signaling using an EGFR tyrosine kinase specific inhibitor can attenuate reactive astrogliosis and promote functional recovery after a traumatic SCI.

**Method:**

The astrocyte scratch injury model *in vitro* and the weight-drop SCI model *in vivo* were used as model systems. PD168393 was used to inhibit EGFR signaling activation. Astrocytic activation and phosphorylated EGFR (pEGFR) were observed after immunofluorescence staining and Western blot analysis. The rate of proliferation was determined by immunofluorescence detection of BrdU-incorporating cells located next to the wound. The levels of TNF-α, iNOS, COX-2 and IL-1β in the culture medium under different conditions were assayed by ELISA. Western blot was performed to semi-quantify the expression of EGFR/pEGFR, glial fibrillary acid protein (GFAP) and chondroitin sulfate proteoglycans (CSPGs). Myelin was stained by Luxol Fast Blue Staining. Cresyl violet eosin staining was performed to analyze the lesion cavity volume and neuronal survival following injury. Finally, functional scoring and residual urine recording were performed to show the rats’ recovery.

**Results:**

EGFR phosphorylation was found to parallel astrocyte activation, and EGFR inhibitor PD168393 potently inhibited scratch-induced reactive astrogliosis and proinflammatory cytokine/mediator secretion of reactive astrocytes *in vitro*. Moreover, local administration of PD168393 in the injured area suppressed CSPGs production and glial scar formation, and resulted in reduced demyelination and neuronal loss, which correlated with remarkable hindlimb motor function and bladder improvement in SCI rats.

**Conclusions:**

The specific EGFR inhibitor PD168393 can ameliorate excessive reactive astrogliosis and facilitate a more favorable environment for axonal regeneration after SCI. As such, EGFR inhibitor may be a promising therapeutic intervention in CNS injury.

## Introduction

With few treatment options, spinal cord injury (SCI) can result in irreversible neurologic deficits influenced by axonal regeneration failure through and beyond the site of injury. The pathogenesis of axonal regeneration failure following SCI involves poor intrinsic central nervous system (CNS) axon regeneration ability as well as the development of an inhibitory growth environment that includes glial scar formation around the injury site, and a major group of inhibitory proteins in and around the scar known as chondroitin sulfate proteoglycans (CSPGs) [1-3].

In response to SCI, astrocytes in the cord become ‘reactive’ and exhibit characteristics including hypertrophy, upregulation of intermediate filament proteins such as glial fibrillary acid protein (GFAP) and vimentin, and cell proliferation [4]. The formation of the inhibitory glial scar environment depends on such properties of reactive astrocytes post-injury. As astrocyte proliferation could result in increased scar formation and elevated CSPG production and deposition near the injury site, it is important to identify mechanisms regulating astrocyte reactive proliferation to potentially enhance axon regeneration and recovery of function after SCI.

Known supportive factors for astrocyte proliferation in CNS injury include growth factors and cytokines [5]. Specifically, epidermal growth factor (EGF) and transforming growth factor-a (TGF-a) have been shown to play crucial roles in the transformation of quiescent astrocytes toward a reactive state [6]. Enhanced expression of the membrane-localized target of these growth factors, the receptor tyrosine kinase epidermal growth factor receptor (EGFR), has been observed in astrocytes following trauma to the CNS [7,8]. Therefore, the EGFR pathway is commonly perceived as a key signaling cascade by which to regulate and control reactive astrogliosis and proliferation following SCI [8]. If so, inhibition of EGFR signaling may benefit axonal regeneration and improve functional outcome in SCI.

PD168393 (4-[(3-bromophenyl) amino]-6-acrylamidoquinazoline) is a potent, cell-permeable, specific and irreversible inhibitor of EGFR [9,10], and acts by covalently modifying cysteine-733, an amino acid located in the catalytic domain of the adenosine triphosphate (ATP) binding pocket [11]. Based on these properties, such a compound would be valuable in investigating the anatomical and functional benefits of inhibiting EGFR post-SCI. To test whether PD168393 could suppress reactive astrogliosis and improve the microenvironment for functional recovery following injury, we used *in vitro* astrocyte scratch injury and *in vivo* weight-drop SCI models to address this issue. Our results show that PD168393 markedly facilitates a more favorable environment for functional recovery after SCI by the amelioration of excessive astrogliosis, which is promising as an effective therapeutic intervention which could be applied in the future to CNS trauma injury.

## Materials and methods

### Spinal cord astrocyte culture

Spinal cord astrocytes from neonatal rat spinal cord were prepared according to previously described methods [12]. Briefly, the spinal cords of one- to two-day-old old rat pups were isolated following painless sacrifice. Meninges were removed and the spinal cord was minced, passed through a 70 mm nylon mesh and then digested in 0.25% collagenase (Sigma, St. Louis, MO, USA) in PBS, pH 7.4, for 10 to 20 minutes at 37°C. Cells were pelleted by centrifugation at 1,000 rpm for two minutes at 5°C and the final pellet was suspended and cells were plated in culture flasks at a density of 1 × 106 to 3 × 106 mL^-1^ and maintained at 37°C in 95% O2/5% CO_2_ in DMEM supplemented with 10% FBS (Hyclone) and 0.5 mg/mL penicillin/streptomycin. The medium was changed 48 hours later and every two days thereafter. During week 2 *in vitro*, non-astroglial cells were removed by shaking [13]. The astrocytes remaining in culture were identified by immunostaining with anti-GFAP. Approximately 95% of the cells were immunopositive for GFAP. All experiments were performed on spinal cord astrocytes maintained for two to three weeks in culture.

### *In vitro* scratch wound model and EGFR inhibitor treatment

A scratch wound model was produced as previously described [14]. Lines were scratched in confluent monolayer astrocytes with sterile plastic pipette tips and then the detached cells and debris were rinsed out with fresh medium PD168393 dissolved in dimethylsulfoxide (DMSO) was added to the cultures after scratch injury at various concentrations (10 μM, 20 μM and 40 μM) (PD168393 group). The final DMSO concentration in the culture medium was 0.2%. A control group of cells and an injured cell group were treated with 0.2% DMSO and not PD168393 in the culture medium. The remaining cell-free area were observed under an Olympus phase contrast microscope (Olympus, Tokyo, Japan) connected to a computer screen and then analyzed with Image-Pro Plus analysis software (Media Cybernetics, Inc., Silver Spring, MD, USA). Cells were harvested at various time intervals (6, 12, 24, 48 and 72 hours) after injury.

### Immunocytochemical staining of cultured astrocytes

The three cell groups were processed for anti-phosphorylated EGFR (pEGFR), GFAP and DAPI (4,6-diamidino-2-phenylindole) immunocytochemical staining at 24 hours post-injury. The cells were fixed in 100% methanol for ten minutes at room temperature and the membranes of fixed cells were permeabilized with Triton X-100. Nonspecific antibody binding was blocked by incubation with 5% BSA in PBS at room temperature for one hour. The cells were then simultaneously incubated with both monoclonal mouse anti-pEGFR (1:100; Abcam, Cambridge, UK) and polyclonal rabbit anti-GFAP (1:500; Sigma, St. Louis, MO, USA) overnight at 4°C. After 3 × 10 minute PBS washes, the cells were incubated in a mixture of two secondary antibodies: FITC-conjugated goat anti-rabbit immunoglobulin G (IgG) antibody (1:200; Pierce, Wilmington, NC, USA) and cyanine 3 (Cy3)-conjugated goat anti-mouse IgG antibody (1:200; Jackson ImmunoResearch, West Grove, PA, USA) for one hour at room temperature. DAPI staining was used to label the nuclei.

In another set of experiments, sister-cultures of astrocyte monolayers were scratched and incubated with 600 ng/mL bromodeoxyuridine (BrdU) (Sigma, St. Louis, MO, USA) to assess cell proliferation. After incubation, the cells were processed for anti-BrdU, GFAP and DAPI immunocytochemical staining at 6, 12, 24 and 48 hours after injury according to a previous protocol [15]. Briefly, after fixation and membrane permeabilization, nuclear proteins were detached from the deoxyribonucleic acid (DNA) by treatment with 2 M hydrogen chloride (HCl) at 60°C for 30 minutes to allow antibody access to the incorporated BrdU. The cells were washed in PBS and blocked in 5% BSA for one hour. The mixture of two primary antibodies used was: monoclonal mouse anti-BrdU (1:100; Sigma, St. Louis, MO, USA) and polyclonal rabbit anti-GFAP (1:500; Sigma, St. Louis, MO, USA). Incubation with the indicated primary antibodies was carried out overnight at 4°C. After being washed in PBS, the cells were incubated in a mixture of two secondary antibodies: fluorescein isothiocyanate (FITC)-conjugated goat anti-rabbit IgG antibody (1:200; Pierce, Wilmington, NC, USA) and Cy3-conjugated goat anti-mouse IgG antibody (1:200; Jackson ImmunoResearch, West Grove, PA, USA) for one hour at room temperature. To determine the proportion of BrdU-positive proliferating cells, the nuclei were labeled with DAPI. Cells were counted next to the wound in five random fields of vision. The results were viewed through a fluorescence microscope (Olympus, BX51, Tokyo, Japan). In control experiments, the primary antibodies were omitted to confirm the specificity of the antibodies.

### ELISA

The levels of TNF-α, induced nitric oxide synthase (iNOS), cyclooxygenase-2 (COX-2) and IL-1β in the conditioned medium from normal cultured astrocytes (control group), scratch wound astrocytes (injury group) and PD168393-treated scratch wound astrocytes (PD168393 group) were assayed by ELISA using Immunoassay Kits (R & D Systems USA) that can detect concentrations as low as 10 pg/ml of the relevant protein, following the manufacturer’s instructions. Briefly, after constructing a standard curve, several dilutions of the test sample were assayed in duplicate for each protein using the appropriate kit. Absorbance was read at 450 nm with a reference wavelength of 570 nm by a VERSA max microplate reader (Molecular Devices, Sunnyvale, CA, USA). Regression analysis was then performed on standards and sample concentrations were determined with reference to the linear portion of the standard curve.

### Animals and surgery

The animal procedures were performed in accordance with protocols approved by the Institutional Animal Care and Use Committee at Nanjing Medical University. Adult female Sprague-Dawley rats (n = 96; weight 220 to 250 g) were randomly classified into SCI and sham-operated groups. A T10 contusion injury was produced as previously described [8,16]. Briefly, rats were anesthetized by intraperitoneal (ip) injection of ketamine (80 mg/kg) and xylazine (10 mg/kg). Once rats were confirmed by toe-pinch to be unconscious, a laminectomy was performed at thoracic level T10. The spine was immobilized stereotaxically, and weight-drop injury was induced using a standardized instrument (New York University Impactor) releasing a weight (10 g, rod diameter of 2 mm) from a height of 12.5 mm on the exposed dura of the spinal cord, inflicting a moderate contusion injury. Following SCI, rats were randomly and blindly assigned to either PD168393 or vehicle treatment groups. Immediately after injury, an osmotic mini pump (Alzet Corp., Palo Alto, CA, USA) placed between the shoulder blades was connected to a 32 gauge catheter and the catheter tip was positioned subdurally on the dorsal side of the spinal cord over the center of the injury through a small hole in the dura mater. In the PD168393-treated group, the osmotic pump was filled 2 mM PD168393 (Sigma, St. Louis, MO, USA) dissolved in 5% DMSO (dissolved in 95% Hank’s balanced salt solution (HBSS)), which was microinfused immediately after pump placement at a rate of 0.5 μl/h for 14 days [7]. Control animals were only given vehicle solution (5% DMSO) lacking PD168393. The overlying back muscles and skin were then sutured. Following surgery, animals were placed and recovered in a warm environment and were later returned to their original housing facility. After 14 days, the pumps were removed and then the wound was closed with surgical suture. Sham-operated animals underwent laminectomy alone. Bladders were expressed twice daily until the animals resumed eating and drinking normally and urinary incontinence disappeared.

### Western blotting analysis

Rats in sham, injury, and PD168393-treated injury groups were deeply anesthetized and sacrificed at day 14 post-injury (n = 3 in each group for each time point). A 15 mm length of the spinal cord containing the injury epicenter was quickly removed for animal tissue protein analysis. For cell culture experiments, cultured astrocytes were harvested at 24 hours after scratch injury. Protein extracts were prepared and Western blotting was performed as described previously [8,17-19]. Samples containing an equal amount of total protein were loaded on SDS-PAGE (10% for EGFR/pEGFR; 12% for other components). After transferring to membrane (300 mA, four hours for EGFR/pEGFR; 250 mA, two hours for others), non-specific binding was blocked by a buffer containing 0.1% Tween 20, 2% BSA, and 5% nonfat dry milk in TBS. Following blocking, the membranes were incubated with primary antibodies diluted in blocking buffer overnight at 4°C. Primary antibodies used for Western blot were monoclonal mouse anti-GFAP (1:1,000; Neomarkers, Fremont, CA, USA) for reactive astrocytes, polyclonal rabbit anti-pEGFR (1:500; Santa Cruz Biotechnology, Santa Cruz, CA, USA), polyclonal rabbit anti-EGFR (1:500; Santa Cruz Biotechnology, Santa Cruz, CA, USA), polyclonal rabbit anti-CS-56 (1:500; Santa Cruz Biotechnology, Santa Cruz, CA, USA) to detect CSPGs and rabbit anti-β-actin (1:2,000; Neomarkers, Fremont, CA, USA) as a loading control. After washing the membranes with 0.1% Tween 20 in TBS, the blots were incubated with horseradish peroxidase-conjugated anti-mouse or anti-rabbit IgG (1:2,000; Neomarkers, Fremont, CA, USA) at room temperature for one hour and visualized with an enhanced chemiluminescence (ECL) system (Thermo Fisher Scientific Inc., USA). The membranes were scanned at 600 dpi, and the digital images were quantitatively analyzed by a Kodak Digital Science 1D system. Optical density (OD) of the signals was semiquantified and expressed as the ratio of OD of the tested proteins to that of β-actin.

### Immunocytochemistry staining of rat spinal cord sections

Rats in all groups were sacrificed at day 14 post-surgery for EGFR, pEGFR, and GFAP double staining (n = 5 in each group), and at day 28 for GFAP, CSPGs and staining (n = 5 in each group), and the tissue was dissected and processed as previously described [8,17]. Briefly, the animals were transcardially perfused with saline, followed by ice-cold 4% paraformaldehyde. The spinal cord was harvested, and a 15-mm length, centered on the injury site, was analyzed. The spinal cord was embedded in Tissue Tek OCT compound (Sakura) and rapidly frozen in nitrogen-cooled isopentane, and stored at −80°C freezer until processing. The tissue blocks were cut at 10 μm thickness, and sections were mounted on poly-L-lysine-coated glass slides and stored at −20°C until further analysis.

Sections were washed in PBS and blocked in 5% normal goat serum for one hour. For immunofluorescence double labeling, sections were treated respectively with a combination of two primary antibodies: mouse anti-GFAP (1:300; Sigma, St. Louis, MO, USA) and rabbit anti-EGFR (1:200; Sigma, St. Louis, MO, USA), mouse anti-GFAP (1:300; Sigma, St. Louis, MO, USA) and rabbit anti-pEGFR (1:200; Sigma, St. Louis, MO, USA), rabbit anti-GFAP (1:300; Sigma, St. Louis, MO, USA) and mouse anti-CSPGs (1:200; Sigma, St. Louis, MO, USA) overnight at 4°C. After 3 × 10 minute PBS washes, sections were incubated in a mixture of two secondary antibodies: Cy3-conjugated goat anti-rabbit IgG antibody (1:300; Jackson ImmunoResearch, West Grove, PA, USA) and FITC-conjugated goat anti-mouse IgG antibody (1:300; Jackson ImmunoResearch, West Grove, PA, USA) for one hour at room temperature. Finally, the slides were washed twice in PBS and once in water for five minutes each before being dried and cover-slipped with antifade mounting media. Sections were observed under an Olympus BX-51 light microscope (Olympus, Tokyo, Japan) connected to a computer screen and then analyzed with Image-Pro Plus analysis software (Media Cybernetics, Inc., Silver Spring, MD, USA).

### Luxol Fast Blue staining and cresyl violet eosin staining

Four weeks post-injury, rats were sacrificed, and the tissue was dissected and processed as stated previously. For myelin quantification, Luxol Fast Blue (LFB) staining (Sigma, St. Louis, MO, USA) was performed on 20 μm thick cross-sections (n = 5 in each group). Cryostat sections were processed according to the cryo-nerve Fast Blue staining kit protocol (GenMed Scientifics Inc. USA). The experimental protocol was described in detail in our previous studies [8,20].

For quantitative analysis of the total lesion volume in whole spinal cords and neuronal survival following injury in all groups, cresyl violet eosin staining was performed on 20 μm thick cross-sections (n = 5 in each group) at the fourth week after SCI. The experimental protocol was performed as previously described [21,22].

Serial sections were used for data presentation. Sections were visualized using an Olympus BX-51 light microscope (Olympus, Tokyo, Japan). An unbiased estimation of the percentage of lesion cavity (or volume of demyelination region) was calculated using previously described methods [22,23]. The percentage total volume of the injured tissue was calculated by dividing the total lesion volume by the total spinal cord volume [22,23]. For rescued motor neuron counting, sections at 1 mm increments rostral and caudal to the epicenter were selected and stained with cresyl violet and eosin. Motor neurons located in the ventral horn (VH) with clearly visible nuclei on both sides of the spinal cord were counted [22].

### Behavioral outcome assessment

Open-field locomotor testing was performed by two trained investigators using the 21-point Basso, Bresnahan, and Beattie (BBB) locomotor scale [24] weekly post-injury. Hindlimb locomotion was then scored from 0 to 21 points (flaccid paralysis to normal gait). Five rats per each experimental group were used for behavioral analysis. All open-field locomotor testing was performed by two blinded examiners under the same conditions on each scheduled testing date.

### Residual urine

After SCI, the urinary function of all rats was disrupted and the bladders were manually expressed twice daily. Urine was collected during bladder expression and volumes were recorded until sufficient (autonomic) bladder function had recovered for at least three weeks [7].

### Statistical analysis

Data are presented as mean ± standard deviation (SD). Statistically significant differences between data (defined as *P* < 0.05) were evaluated by Student’s *t*-test or one-way analysis of variance (ANOVA) followed by Tukey’s *post hoc* test. For comparison between groups over time, a multiple-measurement ANOVA was used.

## Results

### PD168393 inhibited EGFR phosphorylation in reactive astrocytes following a scratch wound

**Figure 1:**
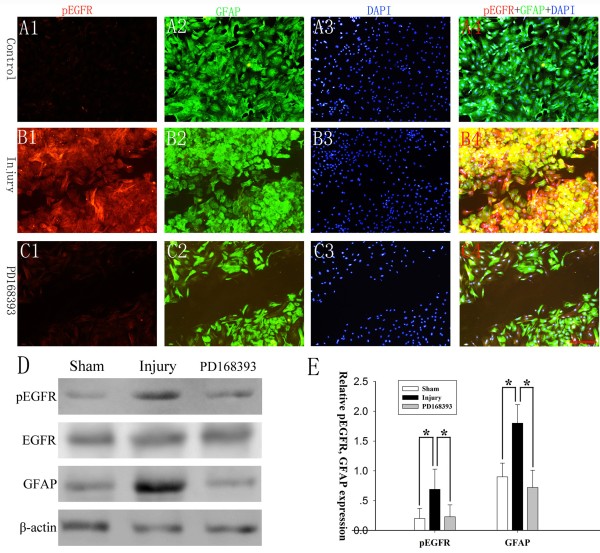
**PD168393 inhibited EGFR phosphorylation of astrocytes in an *in vitro *scratch wound model.** Cultured astrocytes were immunostained for pEGFR, GFAP and DAPI in control **(A1-A4)**, injury, **(B1-B4)** and PD168393 (40 μM) treatment groups **(C1-C4)** at 24 hours after injury (n = 5 in each group). **(A1-C1)** immunolabeling for pEFGR, **(A2-C2)**, GFAP, **(A3-C3)** DAPI and **(A4-C4)** for co-localization of pEGFR, GFAP and DAPI. Scale bars = 100 μm. Representative Western blots of pEGFR and GFAP expression are shown (n = 3/group) **(D)**. Semiquantitative analysis of pEGFR as a ratio of EGFR loading and semiquantitative measurements of GFAP were obtained by normalization to β-actin **(E)**. *P < 0.05.

To investigate whether EGFR phosphorylation increased in scratch-induced reactive astrogliosis *in vitro*, the scratch model was used to determine the effects of injury on astrocyte EGFR phosphorylation. Triple-labeling immunofluorescent for pEGFR, GFAP and DAPI was performed at 24 hours post-injury. The normal cultured astrocytes (control group) barely exhibited EGFR phosphorylation (Figure 1A1-A4). In the injured group, however, most astrocytes showed increased pEGFR immunoreactivity at the ‘wound-edge’ (Figure 1B1-B4), which indicated that a scratch wound could promote astrocyte EGFR phosphorylation. More importantly, EGFR tyrosine kinase specific inhibitor PD168393 administration significantly inhibited pEGFR immunoreactivity in injured astrocytes (Figure 1C1-C4), which provide further evidence that PD168393 could inhibit EGFR phosphorylation in reactive astrocytes. Western blot analysis also supported PD168393 inhibition of EGFR phosphorylation and GFAP expression in reactive astrocytes following injury (Figure 1D-E).

### PD168393 inhibited scratch-induced reactive astrogliosis *in vitro*

**Figure 2:**
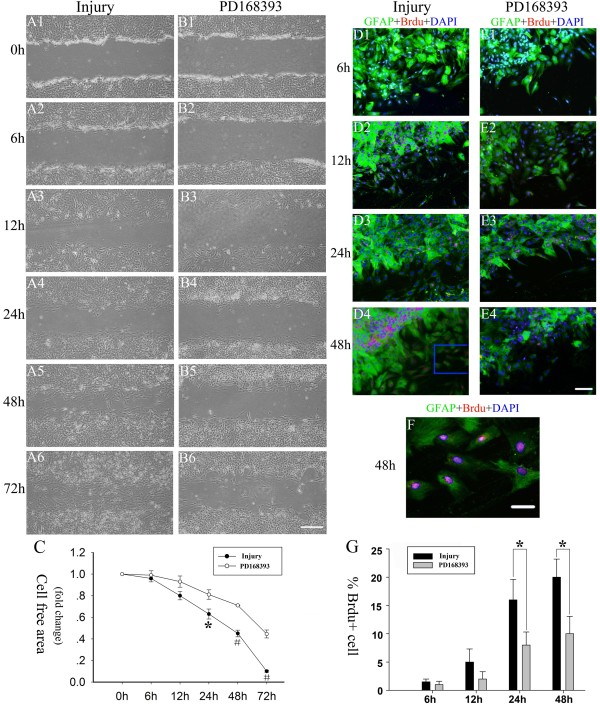
**PD168393 treatment slowed down scratch injury-induced reactive astrogliosis.** Astrocytes were scratched and incubated in the absence or with treatment of 40 μM PD168393. **(A1-B1)**, **(A2-B2)**, **(A3-B3)**, **(A4-B4)**, **(A5-B5)** and **(A6-B6)** are paired images taken from control 0, 6, 12, 24, 48 and 72 hour groups after scratch injury, respectively. Scale bars = 100 μm in **B6** (applies to **A1-B6**). Graphical representation of remaining cell-free area quantification over 72 hours after scratch injury **(C)**. Data are expressed as fold change compared to control group (0 hours). Values are expressed means ± SD (n = 5). Significant difference between time points was observed (*P < 0.05, #*P* < 0.01). Immunostaining for Brdu, glial fibrillary acid protein (GFAP) and 4,6-diamidino-2-phenylindole (DAPI) at 6, 12, 24 and 48 hours after scratch injury in the injury group **(D1-D4)** and PD168393 (40 μM) treatment group **(E1-E4)**, respectively. Astrocytes were identified by GFAP immunostaining (green) and nuclei were identified by DAPI labeling (blue). Astrocytes in S phase were identified by BrdU labeling (red). Insets in panels **(D4)** of co-localization of phosphorylated epidermal growth factor receptor (pEGFR), GFAP and DAPI are shown at high magnification in **(F)**. Scale bars = 100 μm in **E4** (applies to **D1-E4**); scale bars = 20 μm in **(F)**. Percentage of astroglial cells with BrdU labeling at different times after scratch injury **(G)**. *P < 0.05.

To examine the effect of PD168393 on scratch-induced reactive astrogliosis, our scratch wound model was used and the dynamic changes in the morphology, mobility, and proliferation of astrocytes responses to mechanical injury were investigated. Six hours after injury, there were no obvious changes in astrocyte morphology in either the injury or PD168393 group, and cell debris was observed on the surface of cells at the scratch margin (Figure 2A2-B2). Twelve hours post-injury, the astrocytes extended tactile hypertrophic processes towards the denuded area in the injury group (Figure 2A3). However, the hypertrophic changes in the processes of astrocytes in the PD168393 treatment group were less obvious (Figure 2B3). Twenty-four hours after injury, multiple astrocytes with hypertrophic processes as well as many phase-bright spherical somata appeared within the ‘wound-gap’ in the injury group (Figure 2A4), which indicated that activated astrocytes and dividing cells migrated into the denuded area. Interestingly, the cell density of the PD168393 treatment group in the scratch area was smaller than the injury group (Figure 2B4). Forty-eight hours after injury, a near confluent monolayer of activated astrocytes was observed within the wound-gap in the injury group (Figure 2A5). Conversely, few astrocytes were observed in the wound-gap in the PD168393 treatment group (Figure 2B5). In the injury group, seventy-two hours after injury, activated astrocytes with hypertrophic processes gathered into a glial scar-like formation at the wound-gap so that the scratch wound was almost not visible (Figure 2A6). The scratch wound was still distinct, however, in the PD168393 treatment group at this time point as few activated astrocytes were observed at the wound-gap (Figure 2B6). Quantification revealed a reduction in the remaining cell-free area occurred more rapidly in the injury group than the PD168393 group, indicating PD168393 inhibited proliferation of activated astrocytes after injury.

In order to verify the inhibitory effect of PD168393 on astroglial proliferation following *in vitro* injury, we performed double-immunofluorescent labeling for GFAP and BrdU at each designated time point post-injury (Figure 2D-G). BrdU + cell numbers in the wound area were evaluated to determine scratch injury-induced astrocyte proliferation. BrdU is generally considered a marker of dividing cells [25] and is involved in both DNA replication and repair. The number of BrdU + cells significantly increased at 12 hours after injury, and peaked at 24 and 48 hours when the cell density approached confluence in the wound site of injured group (Figure 2D1-D4, F). PD168393 treatment decreased the percentage of BrdU + cells compared to untreated scratch injury cultures. The percentage of BrdU + cells (7.73% ± 2.23%) at the boundary area of the wound at 24 hours was 2.11-fold lower than in untreated cultures (16.31% ± 3.26%). In contrast to the injured group, PD168393 treatment also decreased cell proliferation in areas adjacent to the scratch injury at 48 hours (20.22% ± 3.31% versus 10.03% ± 3.23%) (Figure 2E1-E4, G). These results indicate that PD168393 inhibited proliferation of activated astrocytes following injury *in vitro*.

### Astroglial cell proinflammatory cytokine secretion was reduced by PD168393 following scratch injury *in vitro*

**Figure 3:**
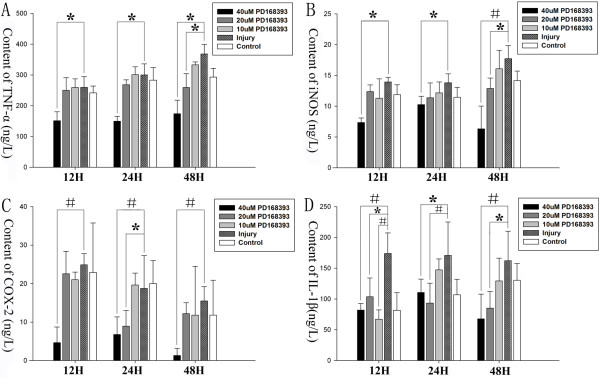
**PD168393 inhibited proinflammatory cytokine/mediators secretion of astrocytes in a scratch wound model.** TNF-α **(A)**, iNOS **(B)**, COX-2 **(C)** and IL-1β **(D)** in conditioned medium from control, injury and PD168393 groups (at 10 μM, 20 μM and 40 μM concentration) were assayed by ELISA at various time intervals (12, 24 and 48 hours after injury). *P < 0.05, #*P* < 0.01.

The concentration of TNF-α, iNOS, COX-2 and IL-1β protein were assayed by ELISA at 12, 24 and 48 hours following scratch injury to assess PD168393 effect on proinflammatory cytokine secretion of astroglial cells after injury. As shown in Figure 3, 40 μM PD168393 significantly decreased the levels of TNF-α, iNOS, COX-2 and IL-1β at three different time points after injury (Figure 3A-D). Twenty micromolar PD168393 treatment also reduced the expression of TNF-α and iNOS at 48 hours after injury (Figure 3A, B). Expression of COX-2 was also inhibited by 20 μM concentration PD168393 treatment at 24 hours after injury (Figure 3C). In addition, IL-1β protein expression was inhibited by 20 μM PD168393 at three different time points post-injury (Figure 3D).

### PD168393 inhibited EGFR phosphorylation in reactive astrocytes following SCI

**Figure 4:**
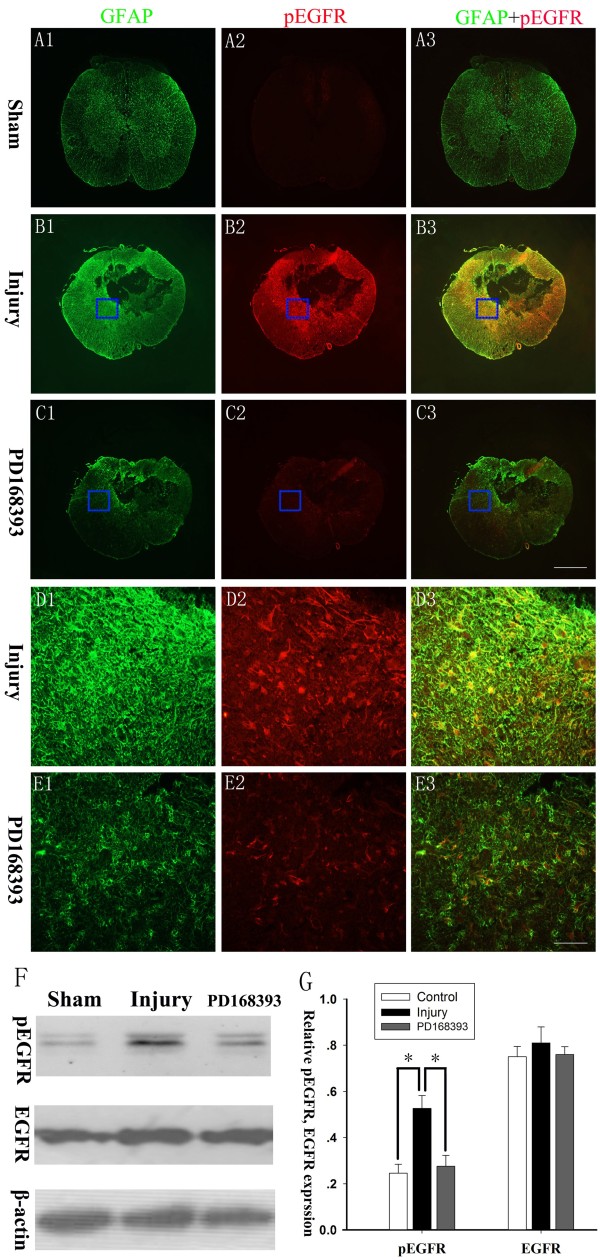
**PD168393 inhibited epidermal growth factor receptor (EGFR) phosphorylation of astrocytes after spinal cord injury (SCI) in rats.** Spinal cord sections were immunostained for phosphorylated EGFR (pEGFR) and glial fibrillary acid protein (GFAP) at two weeks after SCI (n = 5/group). **(A1-C1)** GFAP, **(A2-C2)** pEFGR, and **(A3-C3)** co-localization of pEGFR and GFAP. Insets in panels **(B1-C3)** are shown at high magnification in panels **(D1-E3)**, respectively. Scale bars = 500 μm in **(C3)** (applies to **A1-C3**); scale bars = 50 μm in **(E3)** (applies to **D1-E3**). Representative Western blots of pEGFR and EGFR expression (n = 3/group), and β-actin was a loading control **(F)**. Semi-quantitative measurements were obtained by normalizing to β-actin **(G)**. *P < 0.05.

To determine whether PD168393 could inhibit EGFR phosphorylation in reactive astrocytes following SCI, we performed immunofluorescence double-labeling for pEGFR and GFAP at two weeks following SCI. No EGFR phosphorylation was observed in quiescent astrocytes as almost no co-localization of pEGFR and GFAP was observed in the intact spinal cord (Figure 4A3). A large number of pEGFR and GFAP double-immunofluorescent positive cells surrounded the lesion center in the injured spinal cord (Figure 4B3 and D3), which suggested that EGFR phosphorylation was a common phenomenon in reactive astrocytes post-SCI. Meanwhile, PD168393 administration significantly inhibited pEGFR immunoreactivity in astrocytes (Figure 4C3 and E3). As shown in Figure 4F-G, Western blot results also supported that PD168393 inhibited EGFR phosphorylation in reactive astrocytes following SCI.

### PD168393 attenuated SCI-induced reactive astrogliosis and production of CSPGs

**Figure 5:**
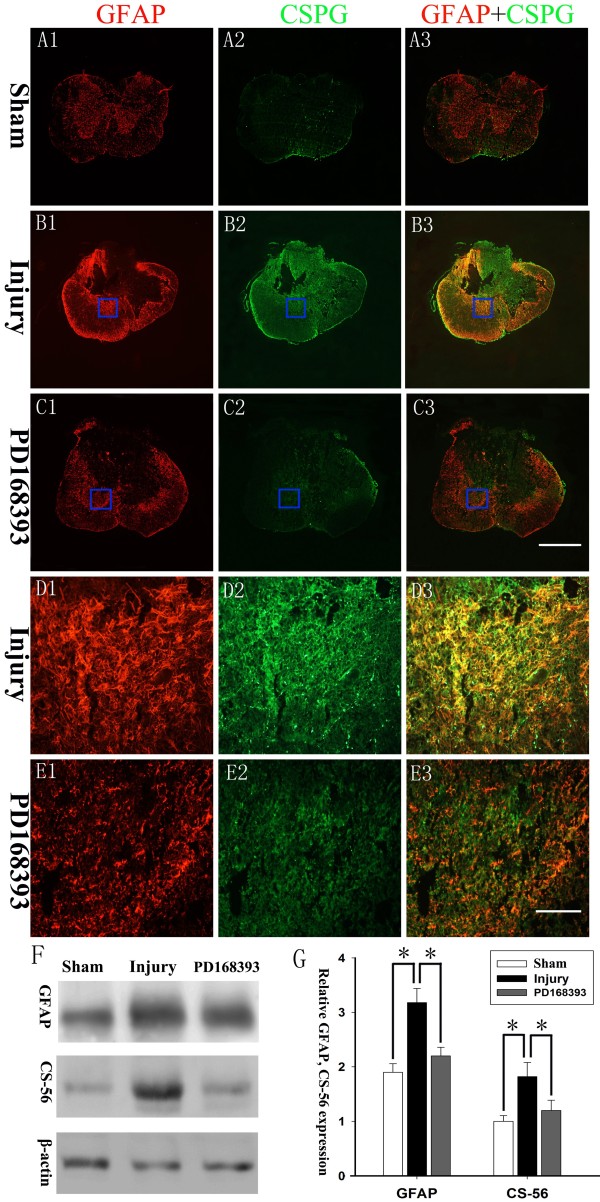
**PD168393 attenuated chondroitin sulfate proteoglycan (CSPG) production from reactive astrocytes after spinal cord injury (SCI).** Double-immunofluorescent labeling for CSPGs and glial fibrillary acid protein (GFAP) at four weeks after SCI (n = 5/group). **(A1-C1)** GFAP, **(A2-C2)** CSPG, and **(A3-C3)** co-localization of CSPG and GFAP. Insets in panels **(B1-C3)** are shown at high magnification in panels **(D1-E3)**. Scale bars = 500 μm in **(C3)** (applies to **A1-C3**); scale bars = 50 μm in **(E3)** (applies to **D1-E3**). Representative Western blots of GFAP and CS-56 expression (n = 3/group), and β-actin was a loading control **(F)**. Semi-quantitative measurements were obtained by normalizing β-actin loading **(G)**. *P < 0.05.

The glial scar consists primarily of reactive astrocytes and heterogeneous extracellular matrix proteins such as CSPGs. To explore the effect of PD168393 on reactive astrogliosis following SCI, immunofluorescence double-labeling for GFAP and CSPG was performed in sham, injury, and PD168393-treated group at day 28 post-injury. As shown in Figure 5B3 and D3, strong and extensive CSPG and GFAP immunoreactivity was detected around the lesion, indicating glial scar formation. More importantly, PD168393 administration not only attenuated astrocyte activation but also inhibited CSPG upregulation at four weeks after SCI (Figure 5C3 and E3). Western blot analysis also revealed similar results (Figure 5F, G).

### PD168393 alleviated demyelination and promoted the survival of VH motor neurons following SCI

**Figure 6:**
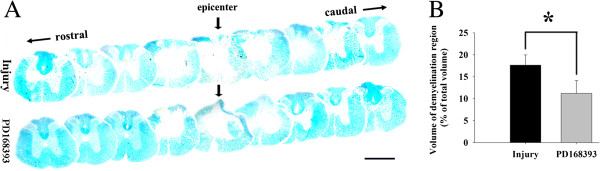
**PD168393 reduced demyelination after spinal cord injury (SCI).** A 15 mm length of spinal cord centered at the injury site was harvested at day 28 post-injury and serially sectioned (n = 5/group). Representative transverse Luxol Fast Blue stained sections at the epicenter and in 1 mm increments rostral and caudal to the epicenter. Epicenter sections are indicated by arrows. Scale bar = 1 mm **(A)**. Graphical representation shows statistically significant reduction in lesion cavity volume following PD168393 treatment as well as considerable decrease in myelin loss **(B)** (n = 5, *P < 0.05 compared to control).

Pathology in tissue cross-sections was identified by the following characteristics: areas of normal myelin appeared bright blue, and demyelinated tissue appeared blanched. There was a large unstained area at the lesion epicenter of the spinal cord in the vehicle-treated group, suggesting weight-drop injury resulted in loss of myelin. However, the PD168393-treated group had a significantly reduced blanched demyelinated area at the lesion epicenter of the injured spinal cord (Figure 6A). Quantification of demyelination region volume showed that PD168393 treated animals had a significant reduction in demyelination region volume (Figure 6B), indicating that PD168393 treatment effectively protected against demyelination following traumatic contusive SCI.

**Figure 7:**
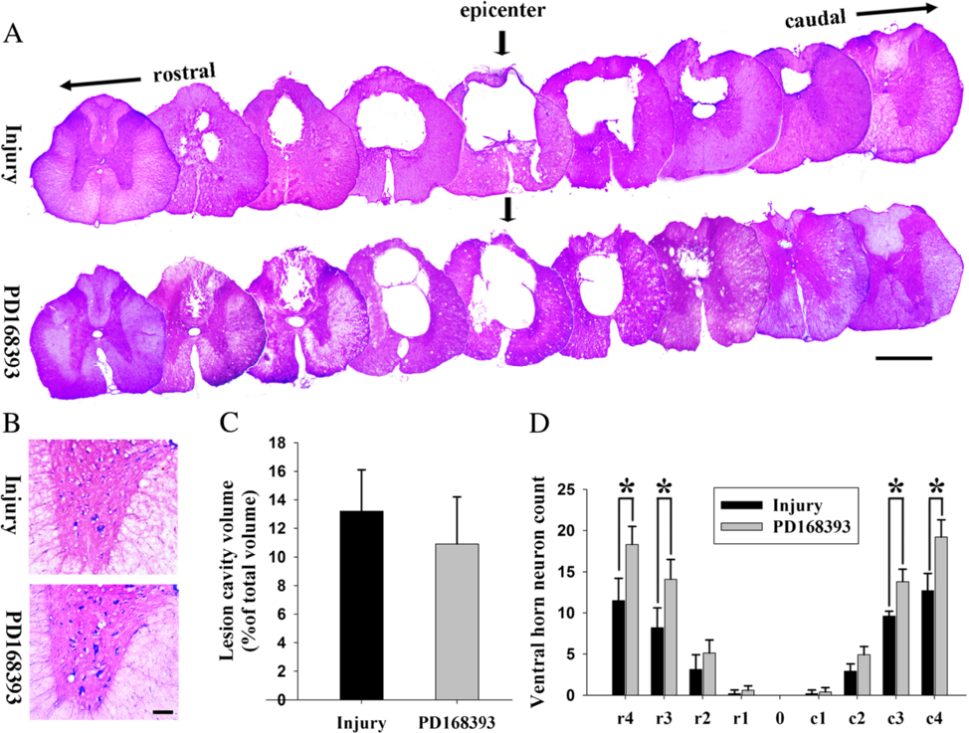
**PD168393 promoted survival of ventral horn (VH) motor neurons after spinal cord injury (SCI).** Representative transverse cresyl violet eosin stained sections of four weeks post-SCI at the epicenter and in 1 mm increments rostral and caudal to the epicenter. Epicenter sections are indicated by arrows. Scale bar = 1 mm **(A)**. Representative photomicrographs from four weeks post-SCI showing cresyl violet eosin stained VH neurons at 4 mm rostral to the injury epicenter. Scale bar = 50 μm **(B)**. Graphical representation showing no statistically significant difference in lesion cavity volume following treatment (n = 5) **(C)**. Comparison of VH neurons among different groups at various distances from the injury epicenter (0) as well as 1 to 4 mm rostral (r) and caudal (c) to it **(D)** (n = 5, *P < 0.05).

Cresyl violet eosin staining photomicrographs showed that there was no different between injury group and PD168393 group in lesion cavity volume, although the demyelinated region volume in the PD168393 group was significantly smaller than the injury group. Also, increased glial scarring around the lesion cavity was observed in the injury group over the PD168393 group. VH motor neurons at the epicenter and in 1 mm increments rostral and caudal to the epicenter (1 to 4 mm) were counted at the fourth week following SCI to determine whether PD168393 treatment promoted neuronal survival following injury. Representative cresyl violet eosin staining photomicrographs showed the motor neurons in the VH (Figure 7B). As quantified in Figure 7C, more residual motor neurons were found in the VH at 3 and 4 mm rostral and caudal to the lesion epicenter in the PD168393 group than in the injury group, which indicated that PD168393 treatment promoted the survival of VH motor neurons following SCI.

### Locomotion and bladder function recovery was improved by PD168393 post-SCI

**Figure 8:**
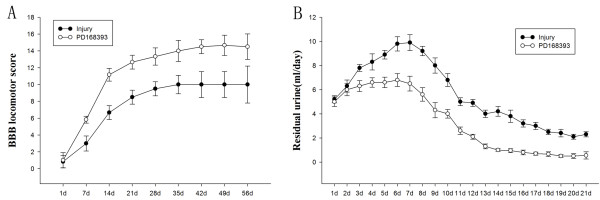
**PD168393 improved functional outcome and bladder recovery after spinal cord injury (SCI).** The Basso, Bresnahan, and Beattie (BBB) scores of hindlimb locomotion were assessed at day 1 post-injury and weekly thereafter. Repeated-measures ANOVA revealed significant difference between injury and PD168393-treated animals (*P* < 0.01) **(A)**. The PD168393-treated rats had less residual urine compared with non-treated injured rats. Statistical analysis between animal groups showed significant differences (*P* < 0.05) **(B)** (n = 5/group).

To assess whether PD168393 could improve functional outcome following SCI, motor function of rats were assessed using a modified BBB hindlimb locomotor rating scale over eight weeks after injury. One day after SCI, all rats exhibited complete bilateral hindlimb paralysis, no significant difference in the extent of locomotor dysfunction between the PD168393 and injured control group was observed. By the first week following SCI, the injury control animals had only hindlimb hip and knee movement corresponding to a BBB score of 3. However, the PD168393-treated animals had extensive movement of three joints of the hindlimb (hip, knee and ankle), corresponding to a BBB score of 6. Recovery in injured control animals plateaued after four weeks with occasional weight supported plantar stepping, and a mean BBB score of 10. In contrast, the PD168393-treated animals continued to improve until week 7 post-injury and showed consistent weight supported plantar stepping with forelimb-hindlimb coordination and an average BBB score of 14 (Figure 8A).

We also recorded residual urine volume of rats every day for three weeks following injury. In vehicle-treated rats, residual urine volumes peaked at seven days, slowly decreased and continued to show significant yields at 21 days after injury; whereas residual urine volumes peaked at four to six days, then rapidly decreased and we documented minimal residual urine volumes from 13 days after injury in the PD168393-treated group. This indicated that PD168393 treatment improved bladder recovery following SCI (Figure 8B).

## Discussion

In the present study, we investigated the effect of specific EGFR inhibitor PD168393 on reactive astrogliosis and proinflammatory cytokine secretion of reactive astrocytes in a scratch injury *in vitro* model and on glial scar formation and microenvironment for axonal regeneration and recovery in a spinal cord contusion rat model. Our results showed that administration of PD168393 markedly inhibited scratch-induced reactive astrogliosis and secretion of proinflammatory cytokines/mediators by reactive astrocytes in culture, as well as suppressing glial scar formation, attenuating myelin loss, promoting the survival of VH motor neurons and more importantly, ameliorating hind limb motor functional and bladder recovery in SCI rats.

The EGF receptors regulate a wide range of cellular processes including proliferation, differentiation, motility, survival, angiogenesis and invasion [8,26]. Our study demonstrated that the up-regulated phosphorylation of EGFR occurred in astrocytes *in vitro* and *in vivo* following injury, and is involved in the pathophysiology of SCI as suggested by previous research [7,8]. Furthermore, EGFR ligands stimulate astrocytes to secrete axon growth inhibitory CSPGs and further contribute to formation of the glial scar [6,27]. Interestingly, our results indicate that administration of EGFR inhibitor PD168393 not only significantly inhibited pEGFR immunoreactivity in astrocytes but also suppressed GFAP expression after SCI. Together, these findings strongly suggest that EGFR activation triggers astrocyte reactivity following injury [6,8].

Reactive astrogliosis is one of the key cellular responses to CNS injury and is considered a major impediment to axonal regeneration [15]. The glial scar is a significant barrier to axonal regeneration, in part because the astrocyte-deposited extracellular matrix (ECM) includes an array of proteoglycans, most notably the CSPGs [28-34]. Many studies have established that CSPGs inhibit neurite outgrowth *in vitro* and restrict axonal regeneration *in vivo*[34-38]. Our data in the present study show that PD168393 administration inhibited EGFR phosphorylation and GFAP expression as well as reduced CSPG expression and attenuated glial scar formation after SCI.

Astrocytes are important in both physiological and pathological processes following SCI and other CNS diseases [39,40], and reactive astrocytes serve as a potential source of inflammatory cytokines [41]. Activated astrocytes can produce the proinflammatory cytokines IL-6, IL-1α and β, TNF-α and interferon γ, and others [34,42,43]. Such properties support a detrimental effect of excessive reactive astrogliosis following SCI. High levels of these cytokines can directly induce neuronal apoptosis and inhibit neurogenesis [44]. Co-activation of proinflammatory cytokines following SCI can also be detrimental to neurons by altering synaptic proteins [45]. In addition, some aspects of the local inflammatory response contribute to spread of secondary injury to potentially viable tissue and lead to delayed apoptotic and necrotic neuronal cell death hours to days after injury [46,47]. Oligodendrocytes are one of the most sensitive glial cells and undergo extensive apoptotic death in response to inflammatory injury. Oligodendrocyte attrition can lead to further demyelination of spared axons and impair their conductive capacity, markedly limiting the recovery of neural function following SCI [8,48]. Our present results indicated that the EGFR inhibitor PD168393 inhibited proinflammatory cytokine secretion from active astroglial cells following injury *in vitro* and protected oligodendrocytes and neurons from death induced by the robust proinflammatory cytokine release following SCI in rats. Therefore, PD168393 may be a promising therapeutic for attenuating inflammatory damage following SCI.

Microenvironmental conditions are important for neuronal survival, neurogenesis and neuron repair [49,50], and the subtle change to this environment by cytokine secretion is involved in pathological processes after SCI [51]. The present study suggested that PD168393 could improve the microenvironment following SCI by suppressing reactive gliosis, proinflammatory cytokines/mediator release and CSPGs deposit. Ultimately, improved functional outcome was achieved.

However, controversial views on the effects of EGFR inhibitor in SCI have been raised. Erschamber *et al*. showed that local blockade of EGFR within the injured area leads not only to structural but also functional recovery following rat contusive SCI [7]. Alternatively, Sharp *et al*. reached an opposite result after repeating these key experiments [52]. The discrepancies between these two studies may be explained by different conditions in animal model procedures, drug delivery manner and data processing methods [53]. The present study showed that EGFR phosphorylation was increased mostly in reactive astrocytes both *in vitro* and *in vivo*. Inhibiting EGFR phosphorylation by PD168393 reduced reactive astrogliosis and CSPGs accumulation and proinflammatory cytokine/protein secretion. Thus, PD168393 ameliorating excessive astrogliosis after injury can mediate through blocking EGFR in activated astrocytes. Meanwhile, another EGFR family member ErbB2 receptor is also associated with astrocytic proliferation [54-56]. Severe gliosis exhibits consistently expressed ErbB2, although normal, quiescent astrocytes and low-grade reactive astrogliosis processes are not associated with significant ErbB2 expression [56]. Since PD168393 also acts as an inhibitor of ErbB2 phosphorylation [10,57], it cannot be excluded that the impairment of astrogliosis by EGFR inhibitor may be mediated through blocking ErbB2 receptor signaling.

Several other studies have explored the potential mechanism by which EGFR inhibitor can promote spinal cord damage axon regeneration. Qu *et al*. showed that EGFR inhibitor suppressed microglia activation and neuroinflammation-associated secondary damage and promoted neuroprotection in rats post-SCI [18]. Microglial activation plays an initial role and is also necessary in maintaining astrogliosis following SCI [58]. Our study provides another perspective on the ability of EGFR blockade to suppress reactive gliosis in SCI. EGFR inhibition by PD168393 has been shown to manipulate glial progenitors to generate neurons but not glia in a SCI model [8], which indicates that EGFR inhibition can suppress the number of proliferative astrocytes post-injury. Douglas *et al*. observed that EGFR inhibitors promote neurotrophin production in glial cells, however the mechanism of this phenomenon is still unknown [59]. These studies provide proof-positive evidence that EGFR inhibition can improve outcomes from experimental SCI; however, the mechanisms of the benefits from EGFR inhibition are still debated.

## Conclusions

In summary, the present study shows that intervention with PD168393 markedly inhibited scratch-induced reactive astrogliosis and proinflammatory cytokines/mediator secretion of reactive astrocytes *in vitro*. More importantly, local administration of PD168393 to the injured rat spinal cord suppressed glial scar formation, attenuated myelin loss, and ameliorated hind limb motor function and bladder recovery in SCI rats. Our results demonstrate that attenuation of astrogliosis, reduction of CSPG expression, suppression of proinflammatory cytokine production and decreasing myelin loss may be potential key mechanisms of EGFR inhibitor effect on improving outcome after SCI. The robust effect of EGFR inhibitor on promoting functional recovery in a rat SCI model could make such compounds particularly attractive candidates for clinical treatment of SCI.

## Abbreviations

ANOVA: analysis of variance; ATP: adenosine triphosphate; BBB: Basso, Bresnahan, and Beattie; BrdU: bromodeoxyuridine; BSA: bovine serum albumin; CNS: central nervous system; COX-2: cyclooxygenase-2; CSPGs: chondroitin sulfate proteoglycans; Cy3: cyanine 3; DAPI: 4,6-diamidino-2-phenylindole; DMEM: Dulbecco's modified Eagle’s medium; DMSO: dimethylsulfoxide; ECL: enhanced chemiluminescence; ECM: extracellular matrix; EGF: epidermal growth factor; EGFR: epidermal growth factor receptor; ELISA: enzyme-linked immunosorbent assay; FBS: fetal bovine serum; FITC: fluorescein isothiocyanate; GFAP: glial fibrillary acid protein; HBSS: Hank’s balanced salt solution; HCl: hydrogen chloride; IgG: immunoglobulin G; IL-1β: interleukin-1 beta; iNOS: induced nitric oxide synthase; ip: intraperitoneal; LFB: Luxol Fast Blue; OD: optical density; PBS: phosphate-buffered saline; pEGFR: phosphorylated EGFR; SCI: spinal cord injury; SD: standard deviation; TGF-a: transforming growth factor-a; TNF-α: tumor necrosis factor alpha; VH: ventral horn.

## Competing interests

The authors declare that there is no conflict of interest associated with this study.

## Authors’ contributions

ZWL, JZ and JJL participated in the design of the research. ZWL, LW, JJL, JZ, JJW and QW carried out the experiments, acquired and analyzed the data. JPZ, XQM, GFS and FW provided technical support during the experiments. ZWL and JZ wrote the paper. All authors read and approved the final manuscript.
